# Trend of hypertension morbidity and mortality in Tigray Region from 2011 to 2015, Tigray, Ethiopia

**DOI:** 10.1186/s13104-018-3488-1

**Published:** 2018-06-08

**Authors:** Hadgu Gerensea, Hafte Teklay

**Affiliations:** 1 School of Nursing, College of Health Sciences & Referral Hospital, Aksum University, Axum, Ethiopia; 2 Department of Biomedical Sciences, College of Health Sciences & Referral Hospital, Aksum University, Axum, Ethiopia

**Keywords:** Trend, Hypertension, Morbidity, Mortality, Tigray

## Abstract

**Objective:**

Hypertension in developing countries were not considered as a major concern. This lack of concern can be explained by the health policy and research of the past years, which had its main focus on infectious diseases but this study focuses on 4 years trend of hypertension mortality and morbidity. A facility based retrospective health management information system record assessment was carried out.

**Result:**

Of the total 66,099 cases registered in the Health Management Information system book, 27,601 were males and 38,498 females. Considering the variation in each year, the overall trend of hypertension mortality has decreased from 32 in 2011/12 to 25 in 2014/15. The number of patients died in 2011 was 1.6 times higher than those who died in 2012. The rate admission of in inpatient and outpatient department visit is increasing from 9257 to 23,633 and 9799 to 24,425 respectively with in 4 years. Though there is variation among reports regarding the rate of morbidity and mortality, Tigray region has been improving its health care for hypertensive patients, which results in a decreasing mortality rate while the morbidity is still alarming.

## Introduction

Hypertension is the consistent elevation of a systemic blood pressure above the normal range [1]. Different factors like age, sex, family history, underlying medical and structural status and leading sedentary life can lead to hypertension [2]. Hypertension is again highly associated with the cardiovascular disease, congestive heart failure, renal disease, peripheral vascular disease and many other complications [3]. In underdeveloped nations, such as Africa, their many focus of health care and research is primarily on infectious diseases [4, 5].

There are 333 million people (34.6%) in developed countries and 639 million people (65.73%) in developing countries who suffer from hypertension. This produces an overall magnitude of hypertension of 26.4% of the world population [6, 7]. Hypertension contributes to 7.6 million premature deaths (that is 13.5% of the total cases) and 92 million disability adjusted life years (DALYS) in 2001 [8]. In Sub Saharan Africa the burden of hypertension is increasing, which results in high morbidity and mortality rate caused by its possibly preventable complications [9]. 20 million Africans are suffering from hypertension, the giving rises from 25 to 35% in prevalence of adults aged 25–65 years, this number also increases with age [10]. In Ethiopia, hypertension accounts for 1.4% of all deaths, stated by the Federal Ministry of Health (FMOH) report, which grades it the 7th leading cause of death in 2001 [11].

The distribution of non communicable diseases including hypertension, cancer and diabetes mellitus is nowadays increasing, but still the health policy is fighting against infectious diseases and their outbreak [12, 13].

There was an increasing trend of hypertension with age and time and slightly increased prevalence among non-Hispanic black Americans from a study conducted in USA by the National Health and Nutrition Examination Survey (NHANES) [14]. Even though many trials have been implemented to control hypertension by different stockholders, the incidence and its impact is still rapidly increasing, both in distribution and magnitude [15].

There are limited studies that were previously conducted regarding what the trend of hypertension in the Tigray region or in the whole of Ethiopia. Such research also needs a massive regional or national primary data source that includes the intended variables providing a baseline. To conclude, this study is aimed at assessing the trend of hypertension morbidity and mortality in the Tigray region.

## Main text

### Study area and period

The study was conducted in the Tigray region which covers an area of 109 km^2^ and its elevation is 2084 M above sea level. The Tigray region has 18 hospitals and 170 health centers and a total population of 4316,988 people. The national regional state is divided into five zones [16–18].

### Study design

Secondary data of the Tigray regional health bureau (TRHB) from August 2011 up to August 2015 was taken and analyzed to assess the trend of hypertension morbidity and mortality.

### Sample size

All patients registered in the 4 years duration from public health institutions of the Tigray Region were considered.

### Data collection procedures

A complete checklist and questionnaire was adopted from similar literatures and was modified based on the study context and to make it convenient to collect the data from the health management information system (HMIS) registration. Data of age, sex, morbidity, mortality, year of diagnosis, type of service (out patient or inpatient) retrieved from the database[health management information system (HMIS) registration].

### Data analysis

The data was entered and coded into SPSS version 21 for analysis. Data analysis included descriptive statistics that were used to describe participants’ demo-graphic characteristics and trend of hypertension mortality and morbidity. The trend of hypertension morbidity and mortality was described through the line graph to understand readers clearly and simply.

### Data quality management

A checklist was properly designed and data was extracted by a statistician from the HMIS data source under close supervision to ensure the data’s quality. Every data sheet was checked and evaluated after collection for its completeness.

### Operational definition

#### Outpatient department

Patients who visit the health institution due to hypertension or co-morbid with other diseases (hypertension and other disease at the same time) once.

#### Inpatient department

Patients who admit to the health institution due to hypertension or co-morbid with other diseases once.

#### Hypertension morbidity

Patients who have hypertension either alone or co-morbid with other like renal failure, stoke etc.

#### Hypertension mortality

Death due to hypertension alone or co-morbid with other like stroke, chronic renal failure) once.

### Result

#### Socio-demographic

The study was conducted in all hospitals and health centers of the Tigray national regional state. A total of 66,099 hypertension cases (of which 104 were dead) were reported to the regional health bureau in the four (July 2011/12–July 2014/15) year duration. All of the data was complete and readable; hence none of it is rejected. There were 27,601 males and 38,498 females registered from the HMIS data source. The ratio of male to female hypertensive is 1:1.4. The table below shows the distribution of the participants for different variables (Table 1).

**Table 1:** A table showing the distribution of participants in age, sex and year according the source, Tigray, Ethiopia

Year	Age classification from the source and sex of participants						Total (%)
	Males [frequency (%)]			Females [frequency (%)]			
	≤ 4	5–14	≥ 15	≤ 4	5–14	≥ 15	
2011/12	18 (12.9)	87 (19.5)	3950 (14.6)	45 (15.3)	136 (28.8)	5563 (14.7)	9799 (14.8)
2012/13	18 (12.9)	126 (28.3)	5731 (21.2)	57 (19.4)	128 (27.1)	8108 (21.5)	14,168 (21.4)
2013/14	24 (17.3)	135 (30.3)	7588 (28.1)	67 (22.8)	98 (20.7)	9795 (26.0)	17,707 (26.8)
2014/15	79 (56.8)	97 (21.8)	9748 (36.1)	125 (42.5)	111 (23.5)	14,265 (37.8)	24,425 (37.0)
Total	139 (0.2)	445 (0.7)	27,017 (40.9)	294 (0.5)	473 (0.7)	37,731 (57.1)	66,099

#### Morbidity trend of hypertension in the Tigray Region

**Fig. 1:**
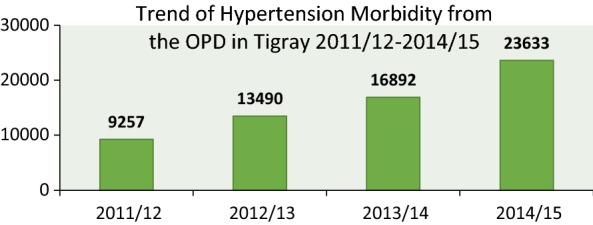
Trend of hypertension morbidity from the OPD, Tigray, 2011/12–2014/15

The 4 years report showed that there are nearly 50 times more patients aged 15 years and above than patients under 15. The magnitude of hypertension morbidity is increasing every year of the 4 years duration; hence it is about 2.5 times higher in 2014/15 compared to 2011/12 (Fig. 1).

**Fig. 2:**
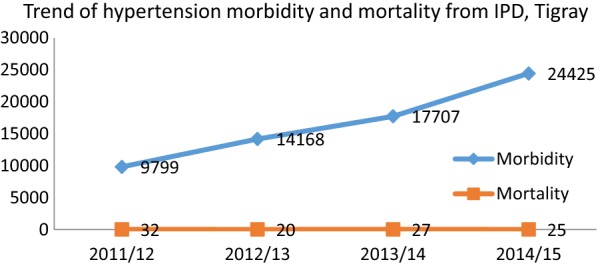
Trend of hypertension morbidity and mortality from IPD and OPD, Tigray, 2011/12–2014/15

In the history of the 4 years duration, the year 2011 showed 9257 clients visiting the outpatient department (OPD) but 2015 showed 23,633 clients which is 2.5 higher. Similarly the magnitude of hypertension inpatient department (IPD) shows 9799 in 2011 and 24,425 in 2015 and visit (OPD) of hypertensive patients is increasing every year in the 4 years duration (Figs. 1 and 2).

#### Mortality trend of hypertension in the Tigray Region

Even though there is intra year variation on the number of deaths and patient registered, generally speaking the trend of mortality is reducing in the Tigray region. The number of patients who died in 2011 was 33 per 10,000 patients and the mortality rate in 2012 was 14 per 10, 000 patients. As there were 9799 patients registered in 2011 and 24, 425 in 2015 with mortality of 32 and 25 respectively, the mortality rate was decreased by 30% from 33 to 10 per 10,000 patients. Finally, the 4 years average fatality rate of hypertension in the region is 18 per 10, 000 hypertensive patients (Fig. 2).

### Discussion

Considering the limitation of the data from the HMIS source registry and the lack of studies regarding the trend, this study has tried to use the all the available data to evaluate the trend of hypertension in the Tigray region. The small number of available studies is due to the HMIS source registry being the only potential source for scientific reports regarding the trends of morbidity and mortality in developing countries like this Ethiopian region.

In the 4 years duration, there was a significant increase in magnitude (from 9257 in 2011/12 to 23,633 in 2014/15) in the morbidity of hypertension in the Tigray region. A study conducted in Addis Ababa confirmed that it is 19.1% prevalent among bank employers and teachers [19]. Recent studies and reports also show that prevalence of hypertension is increasing worldwide and highly increasing in the developing countries. This is comparable with reports from WHO and NHANES [14, 20, 21] in which it is generally stated that the prevalence and its burden of hypertension is alarming.

Trends of hypertension regarding patient admission and visit of outpatient department (OPD) are increasing every year in the current study where the burden of hypertension is high with ages older than 15 years, this is also suggested by other studies [21]. There are tolerable differences in the results of the reports; this is because the current study is conducted based on secondary data and the regional set up might be different relative to the people’s life standard and their awareness to the detection of the disease. Moreover the increase in morbidity from year to year on IPD and OPD in this study may be due to the expansion of health care service, improved health seeking behaviour and improved registration system. Similarly the decreasing mortality is due to improved health care quality and previous patients visit health institution when they are complicated. Furthermore, lifestyle may also contribute.

By 2008, the prevalence of hypertension was the highest in Africa (46%) and the lowest in America (35%), which resulted the global prevalence to be 40% among people of 25 years old and above in the countries surveyed by the WHO. However, the current global adjusted prevalence of hypertension is 26.4% with 333 million affected people (34.6%) in developed countries and 639 million affected people (65.73%) in developing countries [6, 7, 22]. Though there are differences among reports on the projection, a world wide data analysis of a 22 years estimation showed that 1.56 billion (29.1–29.9%) adults in the world will be hypertensive by the year 2025, of which almost three-quarters will originate from economically developing countries [4, 20].

In the current report of 4 years, average of 18 patients out of the 10,000 admitted patients died; this number was accompanied with a decreasing trend of mortality rate every year, because of the improvement of the healthcare. This is relatively comparable to the total Ethiopian deaths from hypertension, which holds that hypertension accounts for 1.4% of all deaths. This makes hypertension the 7th leading cause of death in the country in 2001 [11]. However, this number is lower than the result of a study conducted in Addis Ababa among 3709 adult deaths, which concluded that hypertension is the number one cause of death (12%), followed by a stroke (11%), which again is one of the main complications of hypertension [23, 24].

The reason for this variation could be the difference in the personal characteristics of the study subjects, study design used, the set up for the care and the eligibility of the subjects. Higher rates of mortality were reported from Asia, Europe, and many other countries. Yet, nowadays the dimension of a high rate of mortality caused by hypertension is mainly concerning developing or economically poor nations [8, 21], this may be due to the resources they allocate and the quality of care they provide. It is also reported that death caused by hypertension is estimated to be 7.1 million people out of the total of 13% globally, where the developing nations are faced by a double burden of hypertension and many other cardiovascular diseases [25].

### Conclusion

The study revealed that the patient rate of admission in IPD and OPD visiting is increasing. Hence the trend of hypertension morbidity is increasing every year by 1.4 times; whereas the trend of its mortality is decreasing by 30% from 2011 to 2015 in Tigray region. Though there is variation among reports regarding the rate of morbidity and mortality, the Tigray region has been improving its care for hypertensive patients, which has lead to the mortality rate to decrease, while the morbidity rate is still alarming.

The current health policy is centered around communicable diseases, while non communicable diseases like hypertension should also be put in focus by governmental organizations, non-governmental organizations and policy makers as their burden is booming from time to time with double burden in developing nations, such as the Tigray region.

## Limitation of the study

Data from health facilities are potentially useful for monitoring time trends in the number of HTN cases and deaths but have several limitations. Analysis was based on routinely collected clinical data from public health institution. There is a possibility of both under and over reporting of HTN cases. Since retrospective data were used its accuracy and completeness could not be fully verified. Reporting from facilities to districts and from districts to the ministry of health varies in its completeness and timeliness from institution to institution and this study does not include non-government facilities e.g. private hospitals.

## Abbreviations

HTN: hypertension
IPD: inpatient department
OPD: outpatient department
HMIS: health information management system
TRHB: Tigray regional health bureau
